# Synthesis of phenylazonaphtol-β-D-*O*-glycosides, evaluation as substrates for beta-glycosidase activity and molecular studies

**DOI:** 10.1186/2191-2858-4-2

**Published:** 2014-05-17

**Authors:** Marco Brito-Arias, Carlos Aguilar-Lemus, Pamela B Hurtado-Ponce, Guadalupe Martínez-Barrón, Miguel Ibañez-Hernandez

**Affiliations:** 1 Unidad Profesional Interdisciplinaria de Biotecnología del Instituto Politécnico Nacional, Avenida Acueducto s/n La Laguna Ticomán DF cp 07340, Mexico; 2 Escuela Nacional de Ciencias Biológicas del Instituto Politécnico Nacional, Carpio y Plan de Ayala Colonia Santo Tomas DF cp11340, Mexico

**Keywords:** β-galactosidase, Synthesis, Phenylazonaphtol glycoside, Substrate, Docking

## Abstract

**Background:**

Phenylazonaphtol-β-D-*O*-glycosides are alternative substrates for the detection of enzymatic activity of β-glycosidases which are involved in various important processes. These azoic compounds are currently exploited as prodrugs for colonic disease due the presence of β-glycosidase activity in the gut flora and therefore allowing the release of the drug at the specific site.

**Results:**

Phenylazonaphtol-β-D-*O*-glucoside **3a** and galactoside **3b** were prepared via diazonium salt conditions under weak acidic conditions which do not compromise the *O*-glycosidic bond stability, by coupling reaction between 2-naphtol sodium salt with aminoglycosides **1a** and **1b**. The resulting phenylazonaphtol glycosides **2a** and **2b** were deprotected affording the phenylazonaphtol glycosides **3a** and **3b** in quantitative yield. The galactoside glycoside **3b** was assayed as substrate for *in vitro* β-galactosidase enzymatic activity showing strong absorbance after releasing of the azoic chromophore. Also, docking studies were performed to determine the best pose as well as the interactions between the ligand and the residues located at the active site.

**Conclusions:**

The methodology developed for synthesizing the phenylazonaphtol glycosides described proved to be convenient for generating azoic functionalities in the presence of glycosidic bonds and the glycosides suitable as alternative substrates and potentially useful prodrugs in the treatment of colonic diseases.

## Background

β-glycosidases are hydrolytic enzymes involved in a number of important processes such as defense mechanism, growth regulation [1], gene markers in transgenic plants [2], and prodrugs [3]. The most commonly used substrates for the histochemical localization of β-glycosidases contain the 5-bromo-4-chloro-3-indolyl chromophore attached to most of the known monosaccharides through an *O*-glycosidic bond [4]. After enzymatic hydrolysis, the water soluble indoxyl intermediate must undergo an oxidative dimerization to produce a blue precipitate. However, in some cases before dimerization occurs to produce the indigo dye, some diffusion takes place, producing false positives at some regions in cells lacking enzymatic activity [5]. In addition, phenylazonaphtol glucosides have been synthetically prepared and tested in transgenic plants containing the β-glucuronidase activity which is a widely used gene marker. A comparative test between the commercially available glycosidic indoxyl substrates with the alternative phenylazonaphtol glucuronides resulted in partial diffusion for the indigo dye and no detectable diffusion for the phenylazonaphtol chromophore [6,7].

On the other hand, glycosides when attached to pharmacologically active substances can be used as prodrugs for improving availability, stability, and particularly for specific delivery of the active compound at the target organ. For instance, steroids, antitumor, and anti-inflammatory compounds have been attached to glycosides and evaluated as specific delivery prodrugs [8]. Also, azoic β-glycosides provided to be useful as prodrugs particularly against colitis, Crohn's disease, and colorectal cancer since the delivery at colon level might be achieved by the action of azo-reductase or glycosidase present at the colonic microflora [9].

Due the potential usefulness of phenylazonaphtol glycosides in processes mentioned above, we developed a methodology for preparing phenylazonaphtol glycosides under weak acidic conditions and the resulting azoic glycosides evaluated as β-galactosidase substrates with potential application as prodrugs for colon diseases.

## Methods

**Scheme 1:**
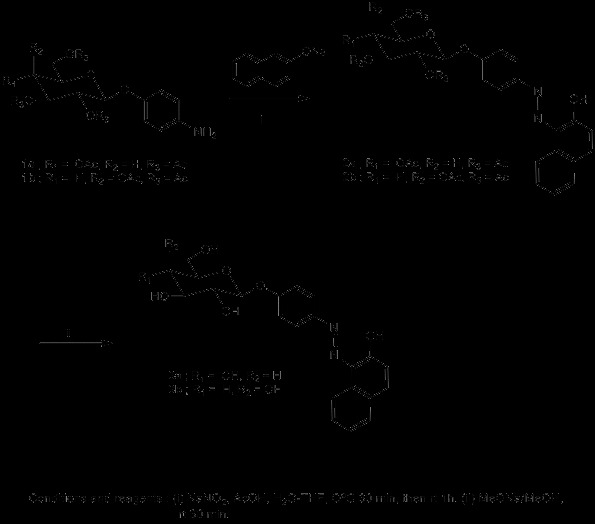
The preparation of the phenylazonaphtol glycosides 3a and 3b The preparation of the phenylazonaphtol glycosides 3a and 3b.

The preparation of the phenylazonaphtol glycosides **3a** and **3b** was achieved according to Scheme 1 consisting in the coupling reaction between 2-naphtol in the sodium salt form with 4-aminophenyl tetracetyl glycopyranoside [10-12]**1a** and **1b** under modified diazonium salt condition, which is mainly the use of acetic acid instead of stronger acids, with sodium nitrite at 0°C to produce the diazonium salts which was condensed *in situ* with 2-naphtol sodium salt providing the corresponding phenylazonaphtol tetracetyl glucopyranoside **2a** and **2b**. Final removal of the acetate protecting groups under Zemplen conditions provided the target phenylazonaphtol glycosides **3a** and **3b** as a reddish solid in 60% yield.

### Scheme

The beta galactosidase assay. β-galactosidase (2 mg; 2.1 U/mg) was suspended in 0.8 mL of buffer containing 0.06 M Na_2_HPO_4_, 0.04 M NaH_2_PO_4_, 0.01 M KCl, 0.001 M MgSO_4_, and 1 mM DTT, pH 7.0, and then incubated with glycoside **3b** (4 mg; 9.3 × 10^-3^ mM) in a mixture of 50 mM acetate buffer with pH 5.0 (0.5 mL) and methanol (0.5 mL) at 37°C for 3 h. The reaction was stopped by adding 0.5 mL 1 M Na_2_CO_3_. The amount of the released phenylazonaphtol chromophore was measured spectrophotometrically at 410 and 455 nm [13].

## Results and discussion

**Figure 1:**
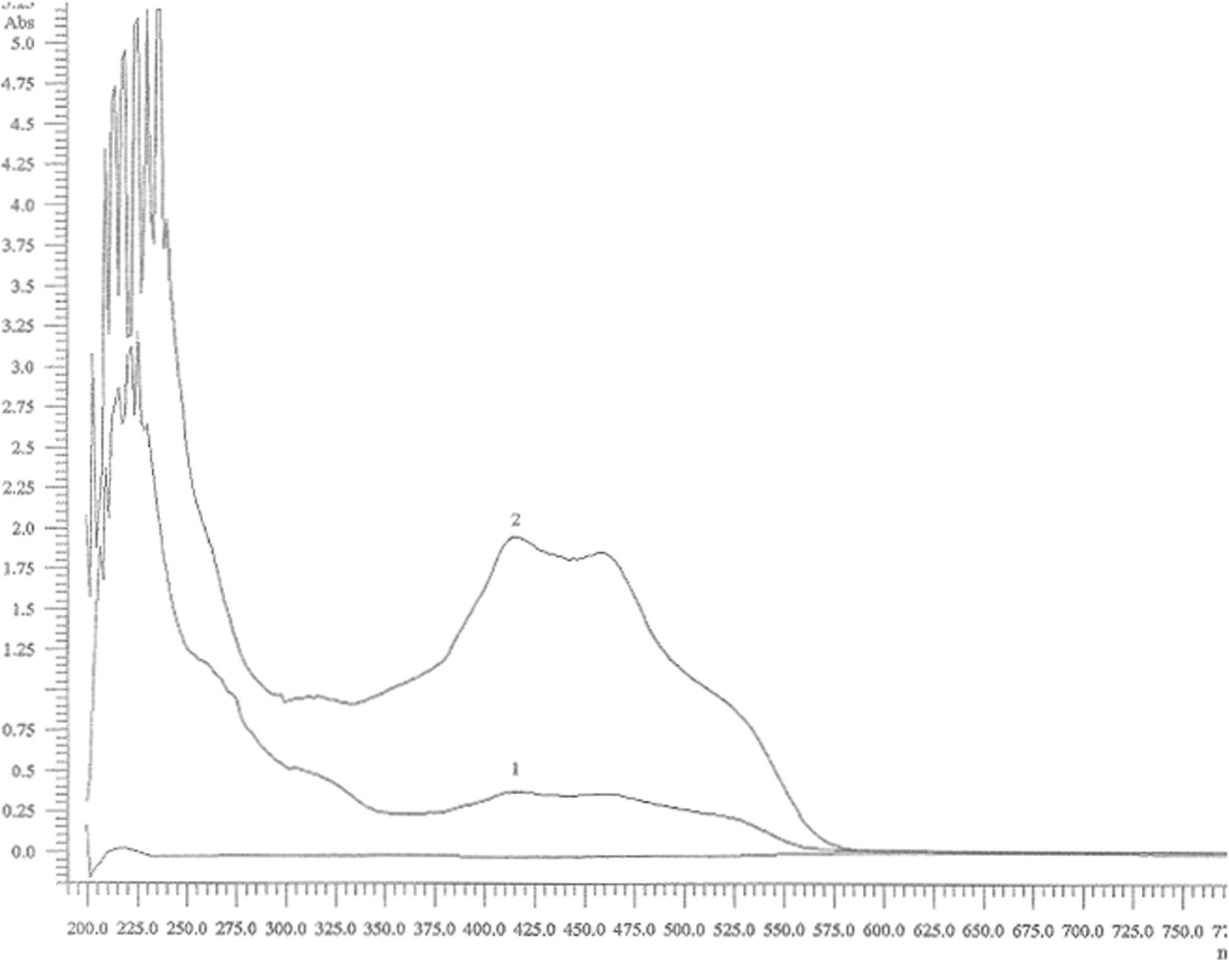
Adsorption of 3b without β-galactosidase (1) and with β-galactosidase (2).

**Figure 2:**
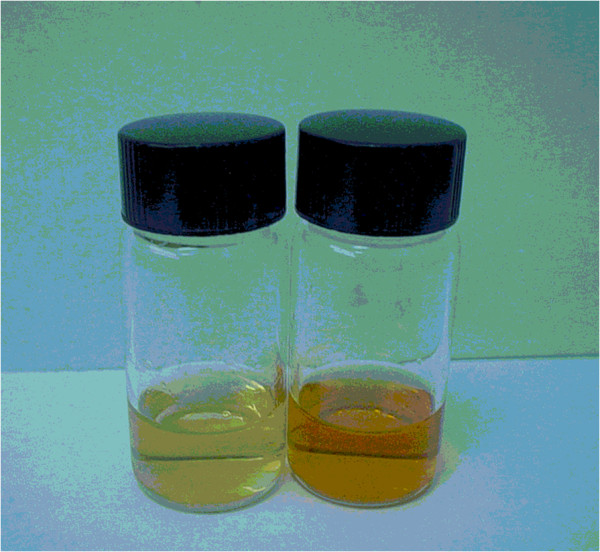
Vials containing 3b without β-galactosidase (less colored) and 3b incubated with β-galactosidase.

The deprotected glycoside **3b** was tested as substrate for the detection of glycosidase activity using commercial *Escherichia coli* β-galactosidase. Thus, the hydrolytic activity resulted in the cleavage of the glycosidic linkage, releasing the phenylazonaphtol chromophore which is observed as an intense reddish color with absorption at 410 and 455 nm, as it is observed in the absorption graphic (Figure 1) and as observed in the vials (Figure 2).

### Docking studies

**Figure 3:**
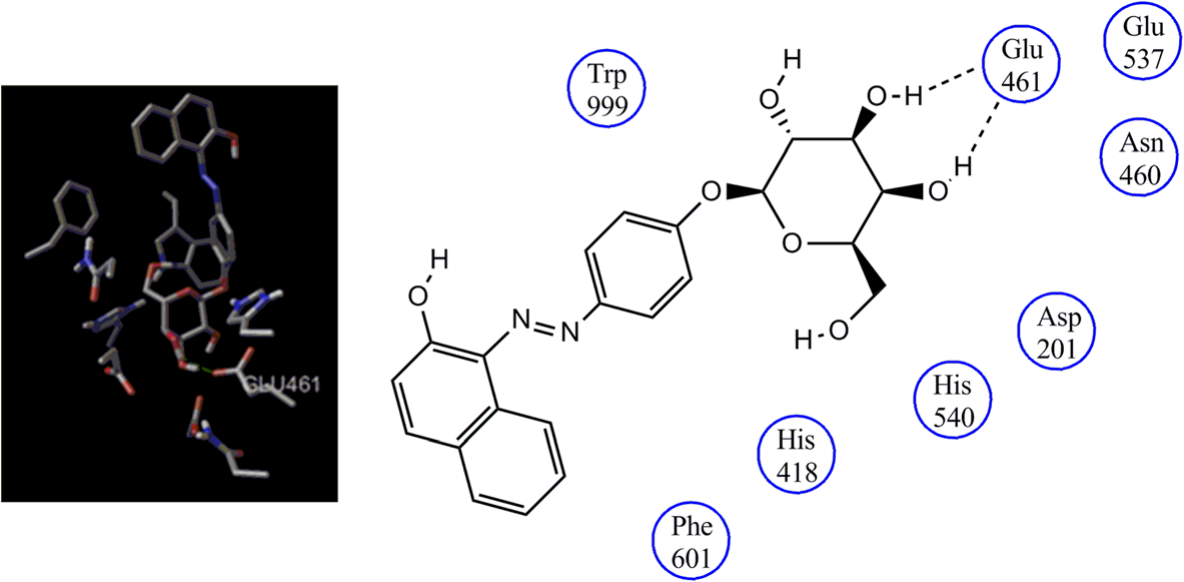
The first scored conformation assumed by 3b presenting hydrogen bond interactions with Glu461 residue.

The active site architecture showing the interactions between the phenylazonaphtol galactoside **3b** and *E. coli* β-galactosidase pocket was performed using AutoDock 4.2 [14] and Chimera [15] software packages as visualization system. The protein assigned for the docking analysis was *E. coli* β-galactosidase (PDB IDs: 1JYN), and the residues chosen were Asn102, Asp201, Asn460, His418, Glu461, Glu537, His540, Phe 601, and Trp999 which have been described to participate in the interactions with its natural substrate lactose [16]. β-galactosidase is a retaining glycosidase with a double displacement mechanism, and its interactions with the ligand proceed with participation of water and the ions Mg^2+^ and Na^1+^ within the catalytic cavity [17]. The docking analysis was performed to find the minimized conformations according to the docking protocols which use the Lamarckian Genetic Algorithm for searching the most favorable ligand-protein complex interactions. Thus, as a result of the docking analysis, we found the minimized conformer with the galactose moiety positioned toward the active site having a binding energy of -11.62, showing hydrogen bond interactions of C-3 and C-4 hydroxyl groups of galactoside moiety with Glu461 residue, in agreement with those reported for the natural substrate lactose [16] (Figure 3).

**Figure 4:**
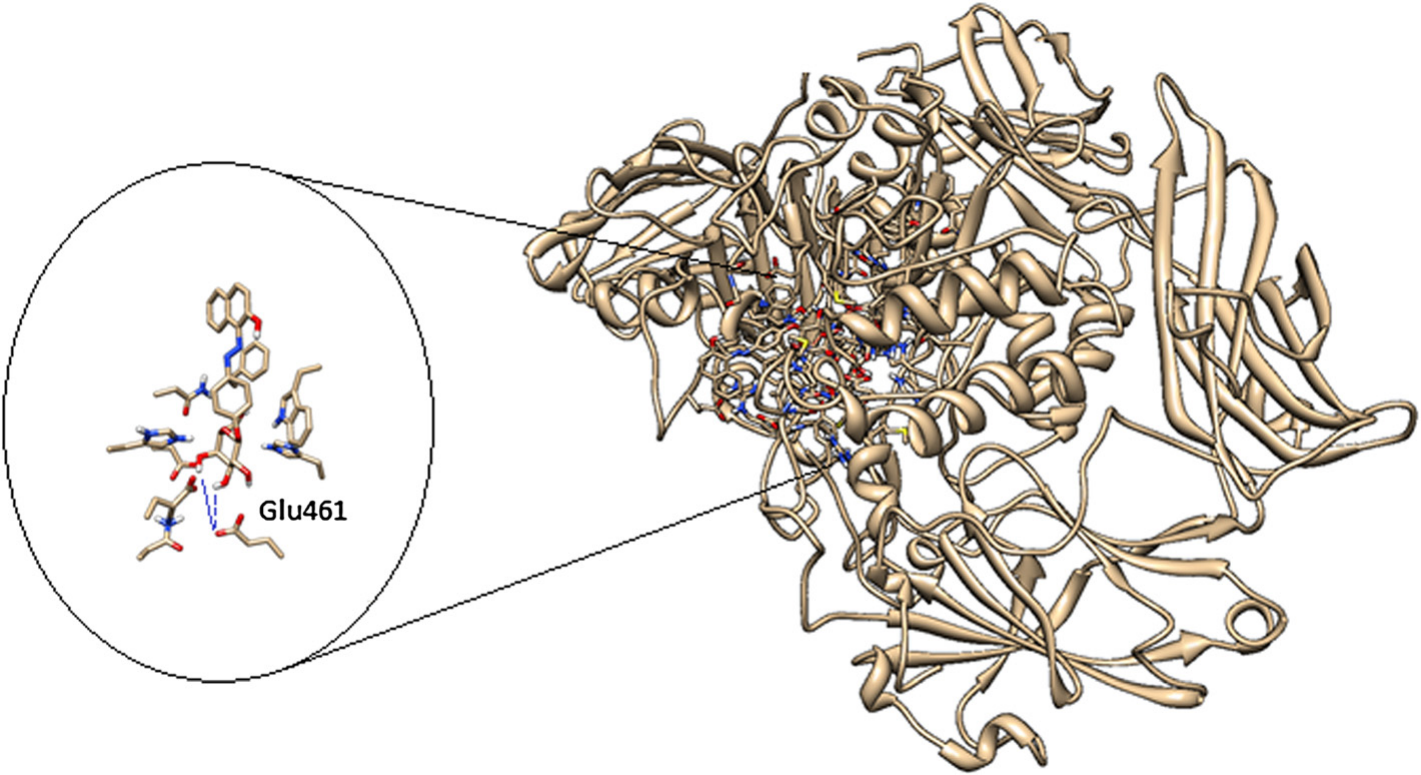
**Ribbon representation.** This shows the region of the active site among the protein and residues involved in the interaction with the ligand.

**Figure 5:**
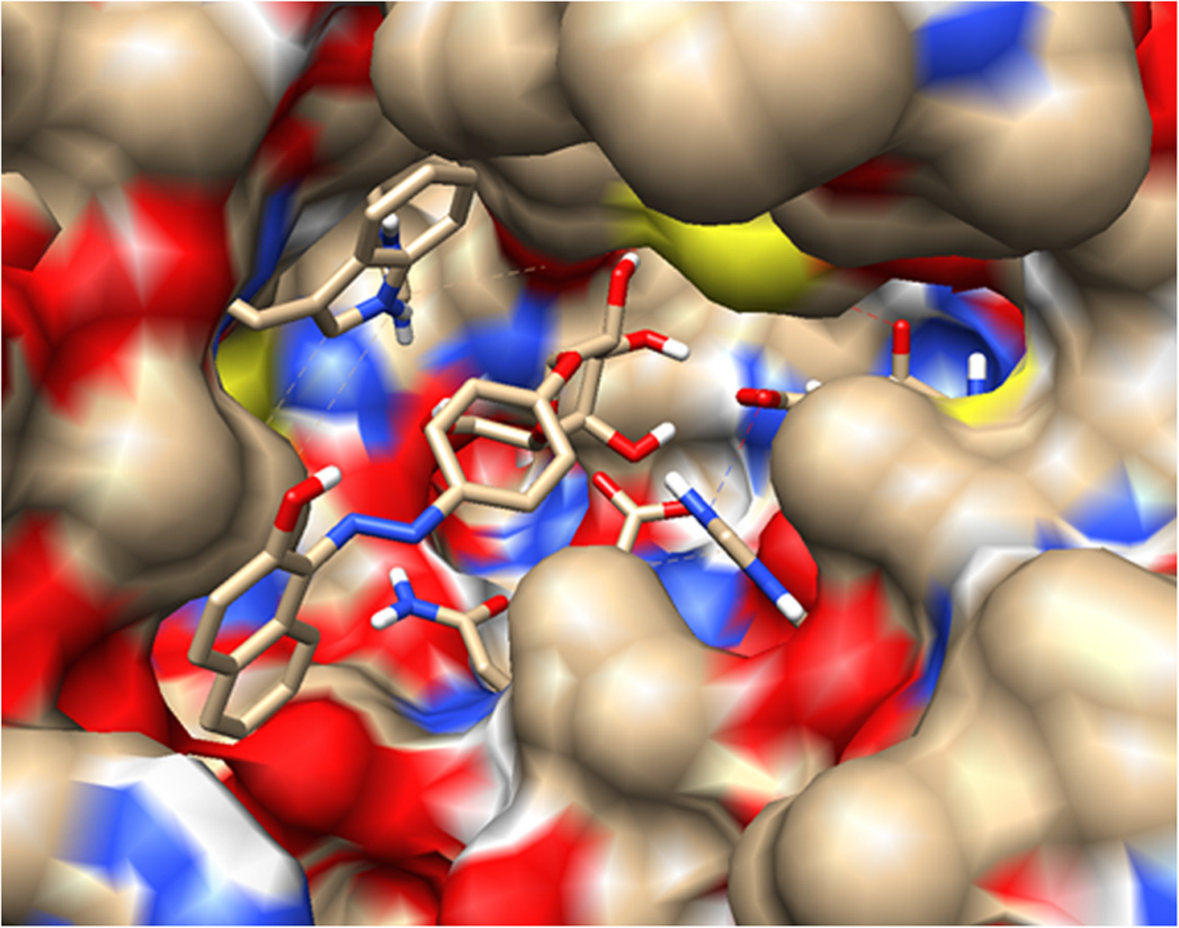
Surface representation showing the pose of ligand 3b at the active.

The ribbon representation was also analyzed by using the chimera visualizing system to show the region of the cavity and the residues involved in the interaction with the substrate **3b** (Figure 4).In addition, the surface representation shows that the galactopyranoside moiety is embedded into the pocket interacting with the residues while the azoic chromophore is pointed out toward the pocket exit as shown in Figure 5.

## Experimental

All the reagents were purchased from Aldrich Chemical Co. (St. Louis, MO, USA) except HBr/acetic acid which was purchased from Fluka Chemical Co. (Buchs, Switzerland). Column chromatography was performed on silica gel with a 70-230 mesh, and thin-layer chromatography on Kieselgel, both from Merck Co. (Darmstadt, Germany), which was used as a detection system. A cerium sulfate solution followed by heating on hot plate. Infrared (IR) spectra were obtained on Perkin-Elmer spectrometer (Waltham, MA, USA); nuclear magnetic resonance (NMR) spectra were recorded on Varian 300-MHz spectrometer (Palo Alto, CA, USA) and Bruker 500 MHz (Karlsruhe, Germany). Mass spectrum (MS) spectra were obtained using a Hewlett-Packard 5989A (Palo Alto, CA, USA). Optical rotations were measured with a Perkin-Elmer 341 polarimeter at 23°C. Enzyme galactosidase was purchased from Sigma Chemical Company (St. Louis, MO, USA).

### 1-[(4-tetra-*O*-acetyl-β-D-glucopyranosyloxy phenyl)azo]-2-naphtol (2a)

**Figure 6:**
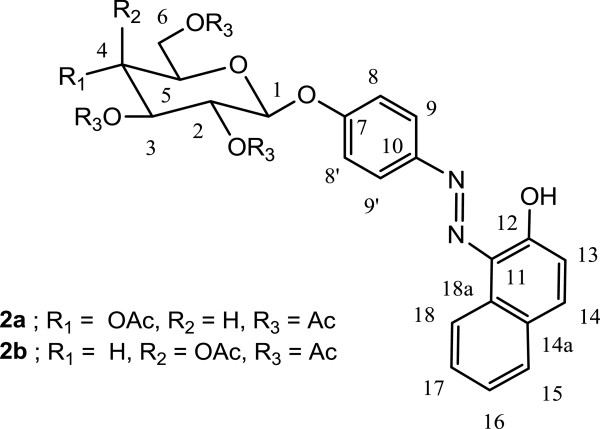
**Structure of 1-[(4-tetra- *O *-acetyl-β-D-glucopyranosyloxy phenyl)azo]-2-naphtol (2a).**

Compound **1a** (0.543 g, 1.23 mmol) was dissolved in 8 mL of THF, and the flask was cooled to 0°C. After the addition of sodium nitrite (85 mg, 1.23 mmol) and 0.5 mL of acetic acid/H_2_O (1:1, *v*/*v*), the reaction was kept under ice-bath temperature with stirring for 15 min. 2-naphtol sodium salt dissolved in 5 mL of THF/H_2_O (1:1, *v*/*v*) was added dropwise in another flask, and the reaction was kept at 0°C with stirring for 30 min, allowing the reaction to reach room temperature and stirred for another 60 min. The solvent was removed under *vacuo*, diluted with CH_2_Cl_2_ (30 mL) and washed with cold 2 N NaOH solution (3 × 15 mL) and water (20 mL). The organic phase was dried over anhydrous sodium sulfate and evaporated on rotavapor. The crude was purified by column chromatography with AcOEt/hexane (1:3) to give an orange-red solid (325 mg, 60%). m.p. 81°C to 83°C, [α]D -13.1 (c 0.6, CHCl_3_), IR (thin film) 1,738 cm^-1^, ^1^H NMR (CDCl_3_) *δ* 2.05 to 2.14 (4 s, 12 H), 3.92 (m, H-5), 4.20 (dd, H-6, *J* = 12.3 Hz, 2.1 Hz), 4.33 (dd, H-6′, *J* = 12.3 Hz, 5.1 Hz), 5.17 (d, H-1, *J* = 7.5 Hz), 5.20 (t, H-4, *J* = 9.6 Hz), 5.31 (t, H-2, *J* = 9.6 Hz), 5.34 (t, H-3, *J* = 9.6 Hz), 6.97 (d, H-13, *J* = 9.3), 7.15 (d, H-8, 8′, *J* = 5.1 Hz), 7.41 (t, H-16, *J* = 7.5 Hz, 7.2 Hz), 7.58 (t, H-17, *J* = 7.5 Hz, 7.2 Hz), 7.66 (d, H-15, *J* = 8.1 Hz), 7.75 (d, H-14, *J* = 9.6 Hz), 7.76 (d, H-9,9′, *J* = 9.0 Hz), 8.62 (d, H-18, *J* = 7.8 Hz). ^13^C NMR (CDCl_3_) *δ* 20.5, 20.6 (4 CH_3_-), 61.8 (C-6), 68.1 (C-4), 71.1 (C-3), 72.1 (C-2), 72.6 (C-5), 98.9 (C-1), 117.8 (C-8), 121.0 (C-9), 121.5 (C-18), 123.0 (C-13), 125.2 (C-16), 127.7 (C-14a), 128.1 (C-11), 128.4 (C-17), 129.7 (C-15), 133.3 (C-18a), 138.2 (C-10), 142.6 (C-14), 156.8 (C-7), 165.1 (C-12), 169.2, 169.3, 170.1, 170.4 (4 C = O). HRMS calculated for C_30_H_30_N_2_O_11_ (M+) 594.1850, found 594.1845 (Figure 6).

### 1-[(4-tetra-*O*-acetyl-β-D-galactopyranosyloxy phenyl azo]-2-naphtol (2b)

Same reaction conditions as for **2a** except that compound **1b** is used instead of **1a**.

^1^H NMR (CDCl_3_) *δ* 2.09 to 2.12 (4 s, 12 H), 4.12 (1H, m, H-5), 4.22 (1H, dd, H-6), 4.26 (1-H, dd, H6′), 5.11 (1H, d, H-1), 5.13 (1H, dd, H-4), 5.48 (1H, t, H-2), 5.54 (1H, dd, H-3), 6.97 (d, H-13, *J* = 9.3), 7.15 (d, H-8, 8′, *J* = 5.1 Hz), 7.41 (t, H-16, *J* = 7.5 Hz, 7.2 Hz), 7.58 (t, H-17, *J* = 7.5 Hz, 7.2 Hz), 7.66 (d, H-15, *J* = 8.1 Hz), 7.75 (d, H-14, *J* = 9.6 Hz), 7.76 (d, H-9,9′, *J* = 9.0 Hz), 8.62 (d, H-18, *J* = 7.8 Hz). ^13^C NMR (CDCl_3_) *δ* 20.8, 20.9 (4 CH_3_-), 61.5 (C-6), 67.0 (C-4), 68.7 (C-2), 71.2 (C-3), 71.4 (C-5), 99.7 (C-1), 117.8 (C-8), 121.0 (C-9), 121.5 (C-18), 123.0 (C-13), 125.2 (C-16), 127.7 (C-14a), 128.1 (C-11), 128.4 (C-17), 129.7 (C-15), 133.3 (C-18a), 138.2 (C-10), 142.6 (C-14), 156.8 (C-7), 165.1 (C-12), 169.2, 169.3, 170.1, 170.4 (4 C = O). MS (EI) m/z 594.18 [M^+^], 169 (100), 264 (62).

### 1-[(4-β-D-glucopyranosyloxy phenyl)azo]-2-naphtol (3a)

**Figure 7:**
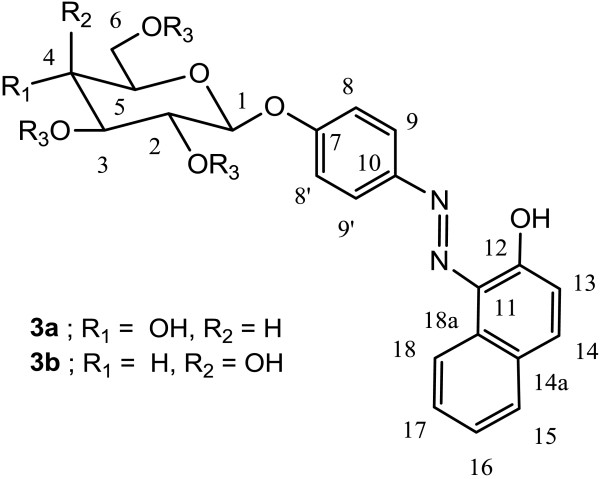
**Structure of 1-[(4-β-D-glucopyranosyloxy phenyl)azo]-2-naphtol (3a).**

To protected phenylazonaphtol glycoside **2a** (0.3 g, 0.504 mmol), 5 mL of 10% NaOMe in MeOH was added and stirred in a room temperature for 2 h. Ion exchange resin Dowex 50WX2-100 was added to neutralize and the resin filtered. The solvent was removed under *vacuo* to give 0.193 g, 90% as a red solid (Figure 7).

m.p. 177°C to 178°C, [α]D -23.4 (c 0.9, MeOH), ^1^H NMR (DMSO d-6) *δ* 3.13 (H-2), 3.30 (H-4), 3.35 (H-5), 3.37 (H-3), 3.60 (H-6), 3.75 (H-6′), 4.99 (H-1, *J* = 7.8), 6.97 (d, H-13, *J* = 9.3), 7.15 (d, H-8, 8′, *J* = 5.1 Hz), 7.41 (t, H-16, *J* = 7.5 Hz, 7.2 Hz), 7.58 (t, H-17, *J* = 7.5 Hz, 7.2 Hz), 7.66 (d, H-15, *J* = 8.1 Hz), 7.75 (d, H-14, *J* = 9.6 Hz), 7.76 (d, H-9,9′, *J* = 9.0 Hz), 8.62 (d, H-18, *J* = 7.8 Hz). ^13^C NMR (DMSO d-6) *δ* 61.7 (C-6), 76.8 (C-5), 71.6 (C-4), 74.1 (C-2), 76.7 (C-3), 96.7 (C-1), 117.8 (C-8), 121.0 (C-9), 121.5 (C-18), 123.0 (C-13), 125.2 (C-16), 127.7 (C-14a), 128.1 (C-11), 128.4 (C-17), 129.7 (C-15), 133.3 (C-18a), 138.2 (C-10), 142.6 (C-14), 156.8 (C-7), 165.1 (C-12), 169.2, 169.3, 170.1, 170.4 (4 C = O). HRMS calculated for C_22_H_22_N_2_O_7_ (M + H) 427.1505, found 427.1509.

### 1-[(4-β-D-galactopyranosyloxy phenyl)azo]-2-naphtol (3b)

Same reaction conditions as for **3a** except that compound **2b** is used instead of **2a**. ^1^H NMR (DMSO d-6) *δ* 3.41 (t, H-2), 3.56 (dd, H-3), 3.61 (m, H-5), 3.62 (H-6), 3.70 (H-6′), 3.84 (dd, H-4), 4.98 (d, H-1, *J* = 8.0), 6.97 (d, H-13, *J* = 9.3), 7.15 (d, H-8, 8′, *J* = 5.1 Hz), 7.41 (t, H-16, *J* = 7.5 Hz, 7.2 Hz), 7.58 (t, H-17, *J* = 7.5 Hz, 7.2 Hz), 7.66 (d, H-15, *J* = 8.1 Hz), 7.75 (d, H-14, *J* = 9.6 Hz), 7.76 (d, H-9,9′, *J* = 9.0 Hz), 8.62 (d, H-18, *J* = 7.8 Hz). ^13^C NMR (DMSO d-6) *δ* 62.0 (C-6), 69.7 (C-4), 72.9 (C-2), 73.8 (C-3), 76.0 (C-5), 97.3 (C-1), 117.8 (C-8), 121.0 (C-9), 121.5 (C-18), 123.0 (C-13), 125.2 (C-16), 127.7 (C-14a), 128.1 (C-11), 128.4 (C-17), 129.7 (C-15), 133.3 (C-18a), 138.2 (C-10), 142.6 (C-14), 156.8 (C-7), 165.1 (C-12), 169.2, 169.3, 170.1, 170.4 (4 C = O). FABMS (M + 1) 427.1.

The Additional file 1 (as word document) shows the ^1^H, ^13^C NMR spectra for the synthesized compounds **2a-b** and **3a-b**, and high-resolution mass spectrometry and elemental analysis are also included.

## Conclusions

The methodology developed for synthesizing the phenylazonaphtol glycosides described proved to be convenient for generating azoic functionalities in the presence of glycosidic bonds, and the glycosides are suitable as alternative substrates and potentially useful prodrugs in the treatment of colonic diseases.

## Competing interests

The authors declare that they have no competing interests.

## Supplementary Material

Additional file 1 **Bidimensional NMR spectroscopy for compound 2a.** Homonuclear through-bond correlation bidimensional spectroscopy (COSY), heteronuclear single quantum coherence (HSQC), and heteronuclear multiple bond correlation (HMBC) experiments are included to provide further evidence for compound **2a.** Click here for file

## References

[B1] Esen Aβ-glucosidase: Biochemistry and Molecular Biology, ACS Symposium Series 533 1993 Washington DC 2 5

[B2] Jefferson RA Kavanagh TA Bevan MW GUS fusions: beta-glucuronidase as a sensitive and versatile gene fusion marker in higher plants EMBO J 1987 6 3901 3907 3327686 10.1002/j.1460-2075.1987.tb02730.x PMC553867

[B3] Tietze LF von Hof JM Krewer B Müller M Major F Schuster HJ Schuberth I Alves F Asymmetric synthesis and biological evaluation of glycosidic prodrugs for a selective cancer therapy Chem Med Chem 2008 3 12 1946 1955 10.1002/cmdc.200800250 19021160

[B4] Manafi M Kneifel W Bascomb S Fluorogenic and chromogenic substrates used in bacterial diagnostics Microbiol Rev 1991 55 335 348 1943991 10.1128/mr.55.3.335-348.1991 PMC372823

[B5] Lodja Z An improved histochemical method for the demonstration of disaccharidases with natural substrates Histochemie 1970 22 347 361 4561400 10.1007/BF00277599

[B6] Terryn N Brito-Arias M Engler G Tiré C Villaroel R Van Montagu M Inzé D rha1, a gene encoding a small GTP binding protein from *Arabidopsis*, is expressed primarily in developing guard cells Plant Cell 1993 5 1761 1769 10.1105/tpc.5.12.1761 8305870 PMC160402

[B7] Van der Eycken E Terryn N Goemans JL Carlens G Nerinckx W Claeyssens M Van der Eycken J Van Montagu M Brito-Arias M Engler G Sudan-b-D-glucuronides and their use for the histochemical localization of b-glucuronidase activity in transgenic plants Plant Cell Rep 2000 19 966 970 10.1007/s002990000219 30754840

[B8] Friend DR Chang GW Drug glycosides: potential prodrugs for colon-specific drug delivery J Med Chem 1985 28 51 57 10.1021/jm00379a012 3965714

[B9] Friend DR Chang GW A colon-specific drug-delivery system based on drug glycosides and the glycosidases of colonic bacteria J Med Chem 1984 27 261 266 10.1021/jm00369a005 6699871

[B10] Ganjian I Basile DV Reductive syntheses of *p*-aminophenyl-β-D-glucoside and its conversion to β-glucosyl Yariv reagent Anal Biochem 1997 246 152 155 10.1006/abio.1997.9974 9056204

[B11] Barni E Barolo C Quagliotto PL Valldeperas J Viscardi G Novel azobenzene derivatives containing a glucopyranoside moiety Dyes Pigments 2000 46 29 36 10.1016/S0143-7208(00)00033-4

[B12] Amaike M Kobayashi H Sakurai K Shinkai S Novel attempts to change the colour of dye molecules utilizing the aggregation mode of saccharides Supramol Chem 2002 14 245 253 10.1080/10610270290026158

[B13] Stiborová M Asfaw B Frei E Schmeiser HH Wiessler M Identification of 1-(3,4-dihydroxyphenylazo)-2-hydroxynaphthalene as the product of oxidation of 1-phenylazo-2-hydroxynaphthalene (Sudan I, solvent yellow 14) by rat liver microsomes Collect Czech Chem Commun 1994 59 2727 2733 10.1135/cccc19942727

[B14] Sanner MF Huey R Dallakyan S Karnati S Lindstrom W Morris GM Norledge B Omelchenko A Stoffler D Vareille G AutoDockTools, version 1.4.5 2007 The Scripps Research Institute, La Jolla

[B15] Pettersen EF Goddard TD Huang CC Couch GS Greenblatt DM Meng EC Ferrin TE UCSF chimera—a visualization system for exploratory research and analysis J Comput Chem 2004 25 13 1605 1612 10.1002/jcc.20084 15264254

[B16] Juers DH Tom D Heightman TD Vasella A McCarter JD Mackenzie L Withers SG Matthews BW A structural view of the action of *Escherichia coli* (lacZ) α-galactosidase Biochemistry 2001 40 14781 14794 10.1021/bi011727i 11732897

[B17] Jancewicz LJ Wheatley RW Sutendra G Lee M Fraser ME Huber RE Ser-796 of b-galactosidase (*Escherichia coli*) plays a key role in maintaining a balance between the opened and closed conformations of the catalytically important active site loop Arch Biochem Biophys 2012 517 111 122 10.1016/j.abb.2011.11.017 22155115

