# Supplementary material for: Synthesis of phenylazonaphtol-β-D-O-glycosides, evaluation as substrates for beta-glycosidase activity and molecular studies
Source: Org Med Chem Lett. 2014 May 17;4:2. doi: 10.1186/2191-2858-4-2 (PMC4074863; doi:10.1186/2191-2858-4-2)
Supplement: Additional file 1 — Bidimensional NMR spectroscopy for compound 2a. Homonuclear through-bond correlation bidimensional spectroscopy (COSY), heteronuclear single quantum coherence (HSQC), and heteronuclear multiple bond correlation (HMBC) experiments are included to provide further evidence for compound 2a. [file 2191-2858-4-2-S1.pdf]

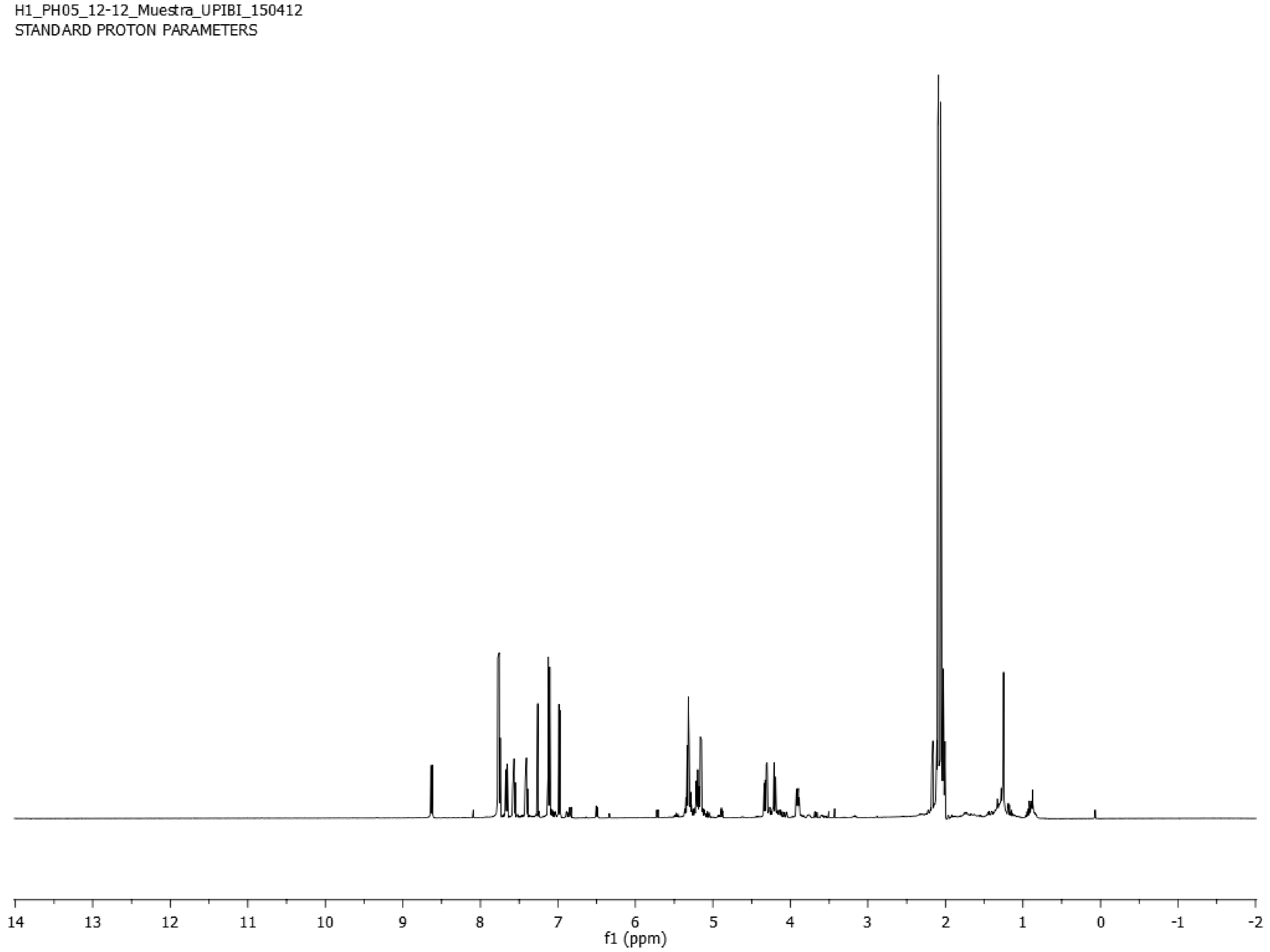
H1_PH05_12-12_Muestra_UPIBI_150412
STANDARD PROTON PARAMETERS
14
13
12
11
10
9
8
7
6
5
4
3
2
1
0
-1
-2
f1 (ppm)

H1_PH05_12-12_Muestra_UPIBI_150412
STANDARD PROTON PARAMETERS

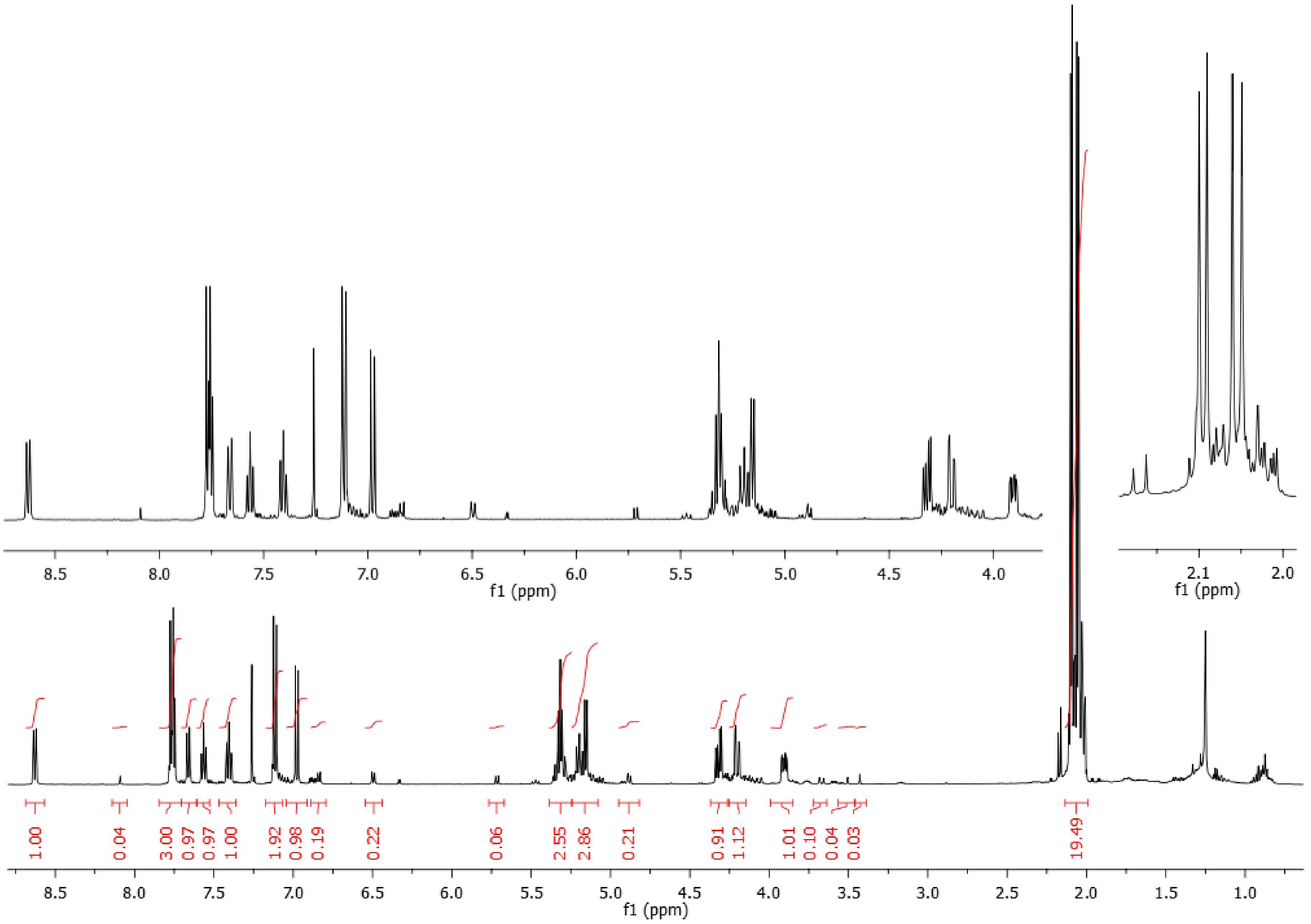

C13_PH05_12-13_150412
STANDARD CARBON PARAMETERS

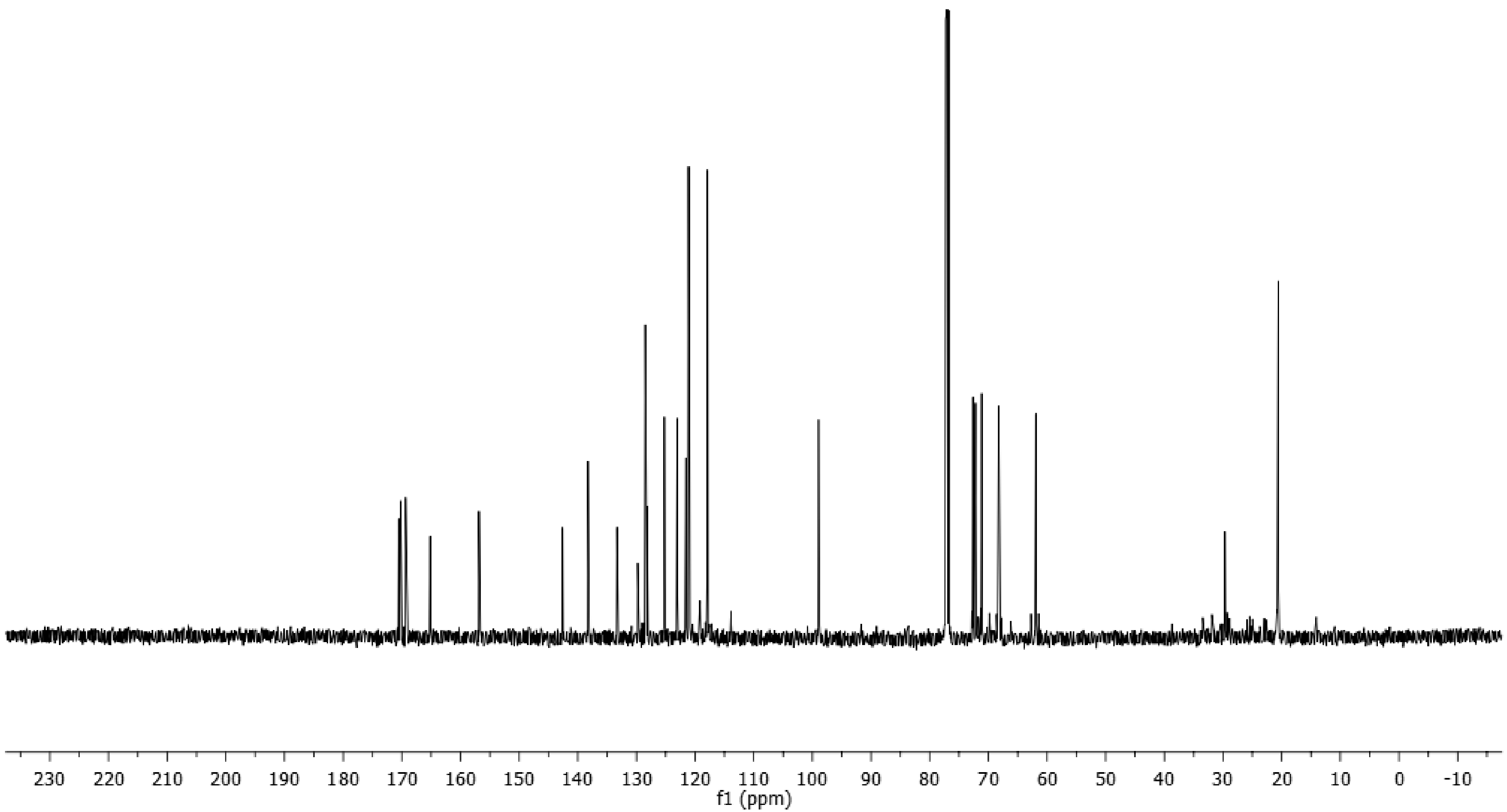

C13_PH05_12-13_150412
STANDARD CARBON PARAMETERS

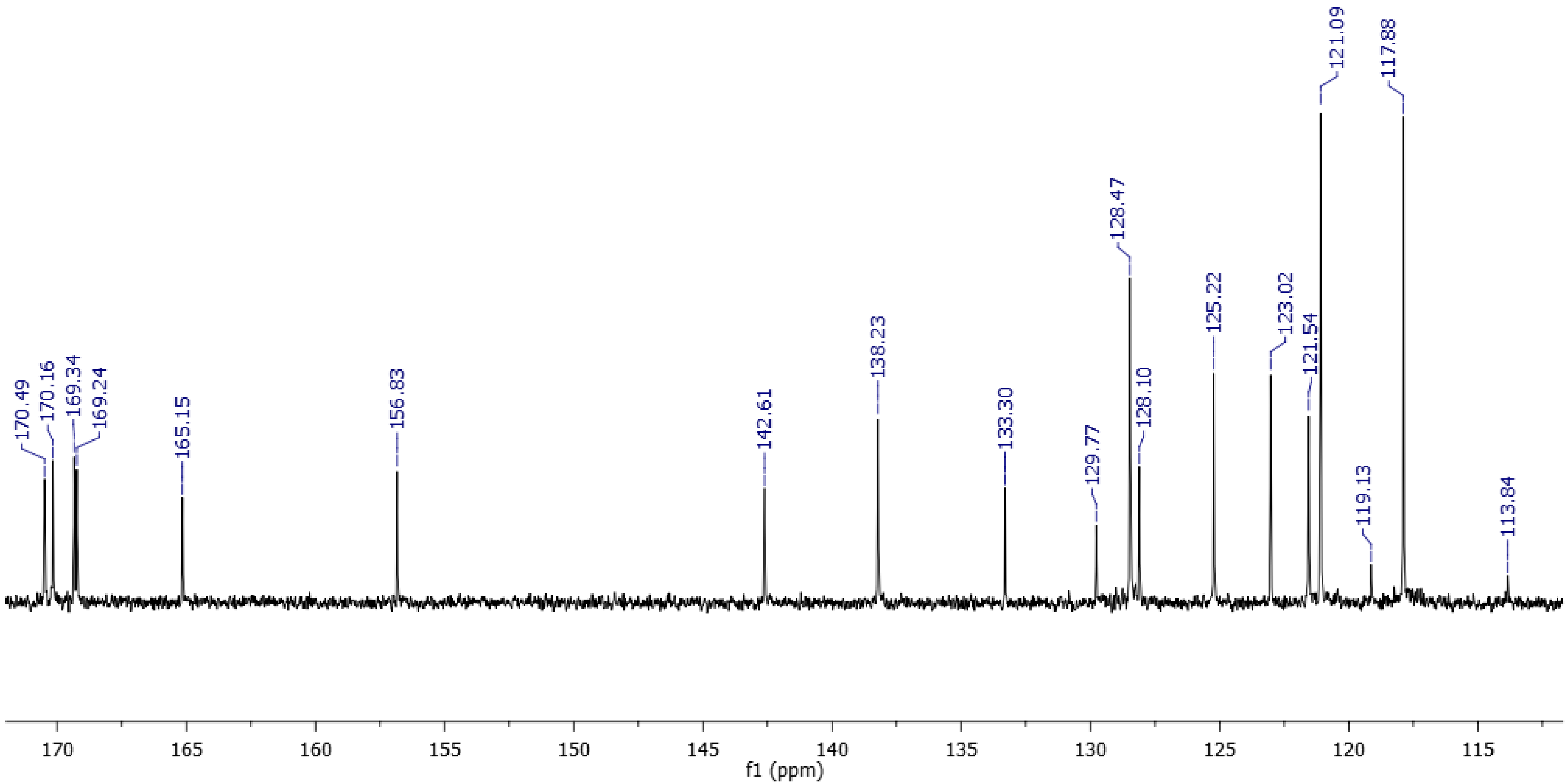

C13_PH05_12-13_150412
STANDARD CARBON PARAMETERS

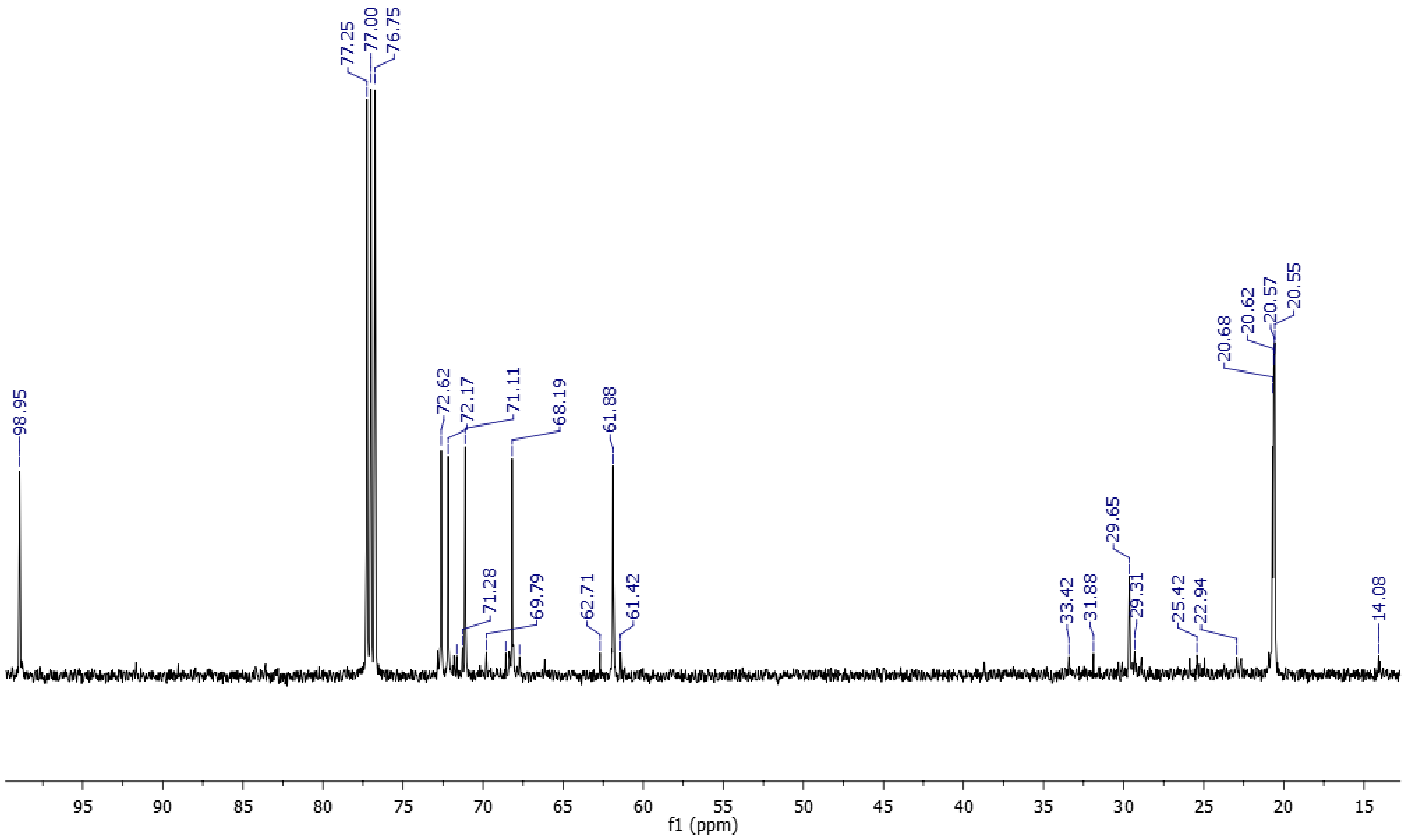

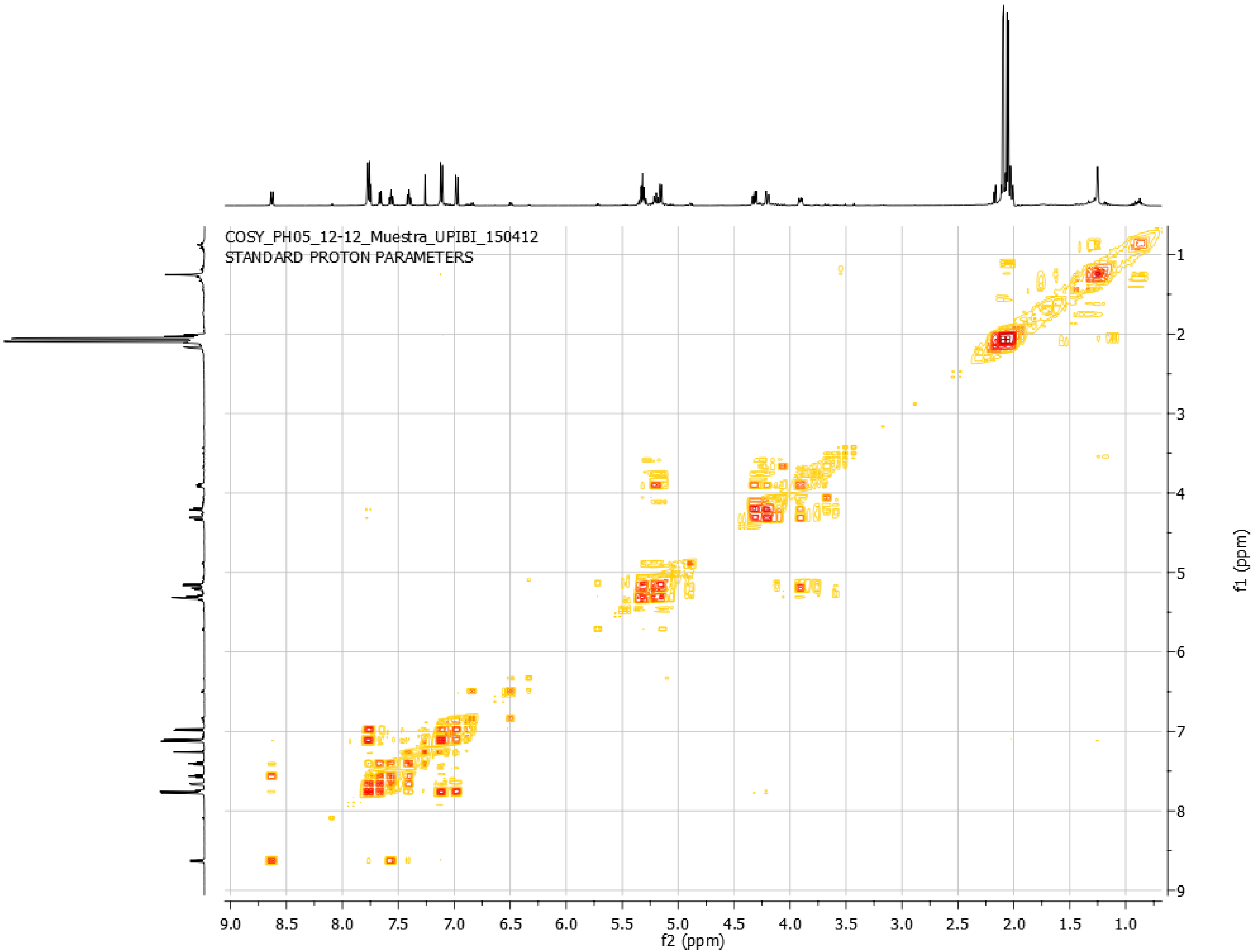

COSY_PH05_12-12_Muestra_UPIBI_150412
STANDARD PROTON PARAMETERS
f2 (ppm)
f1 (ppm)
9.0
8.5
8.0
7.5
7.0
6.5
6.0
5.5
5.0
4.5
4.0
3.5
3.0
2.5
2.0
1.5
1.0
1
2
3
4
5
6
7
8
9

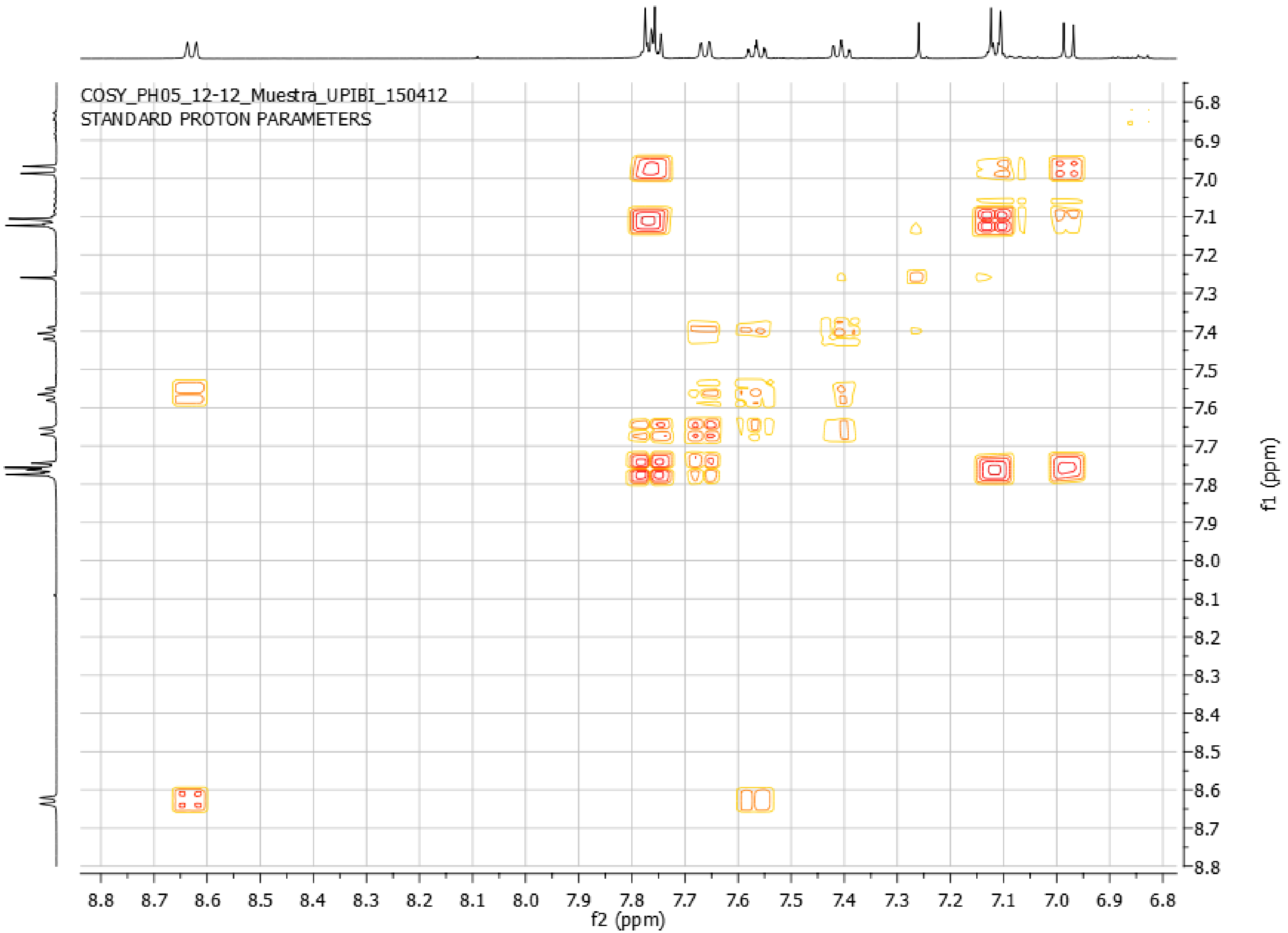

COSY_PH05_12-12_Muestra_UPIBI_150412
STANDARD PROTON PARAMETERS
f2 (ppm)
f1 (ppm)

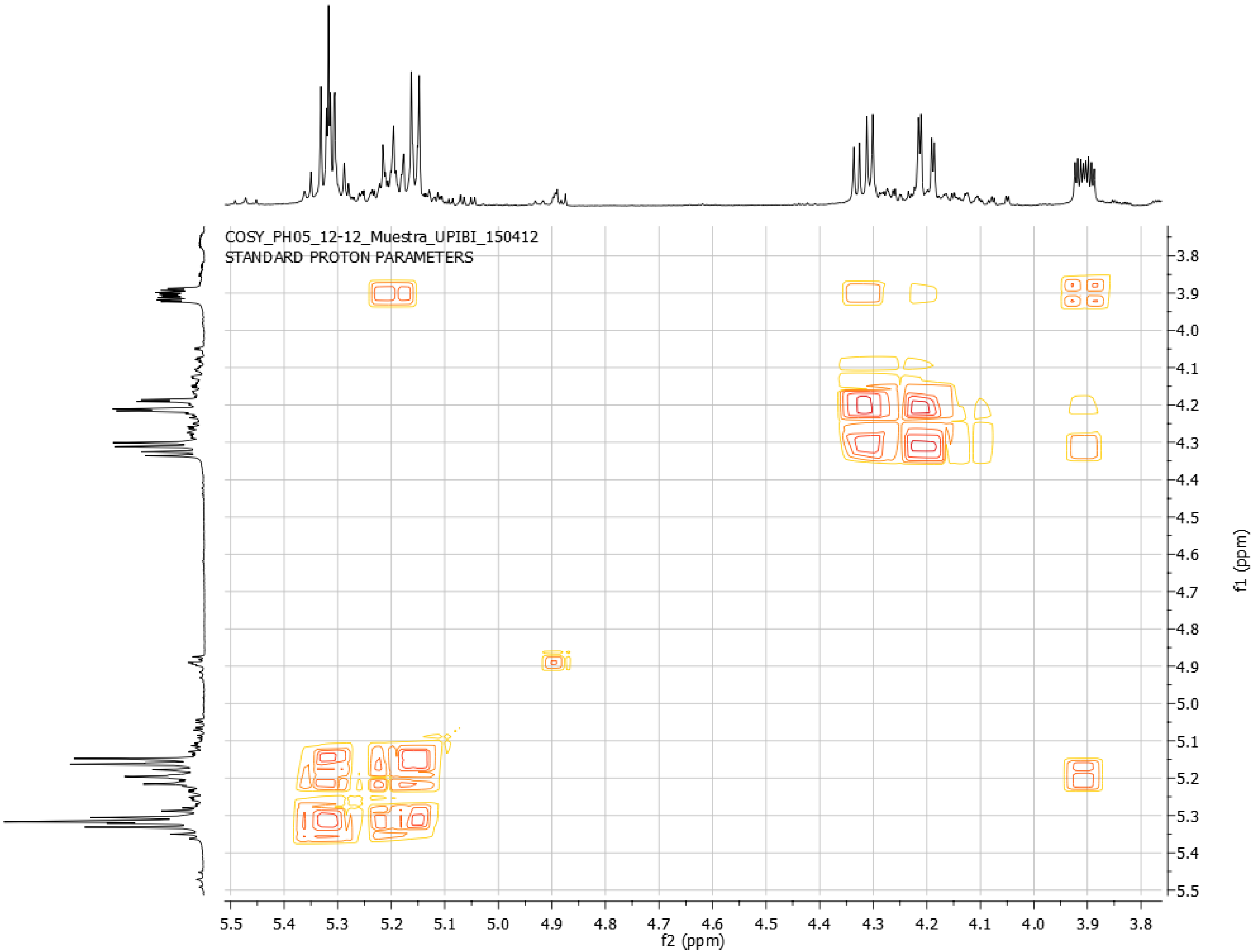

COSY_PH05_12-12_Muestra_UPIBI_150412
STANDARD PROTON PARAMETERS
f2 (ppm)
f1 (ppm)

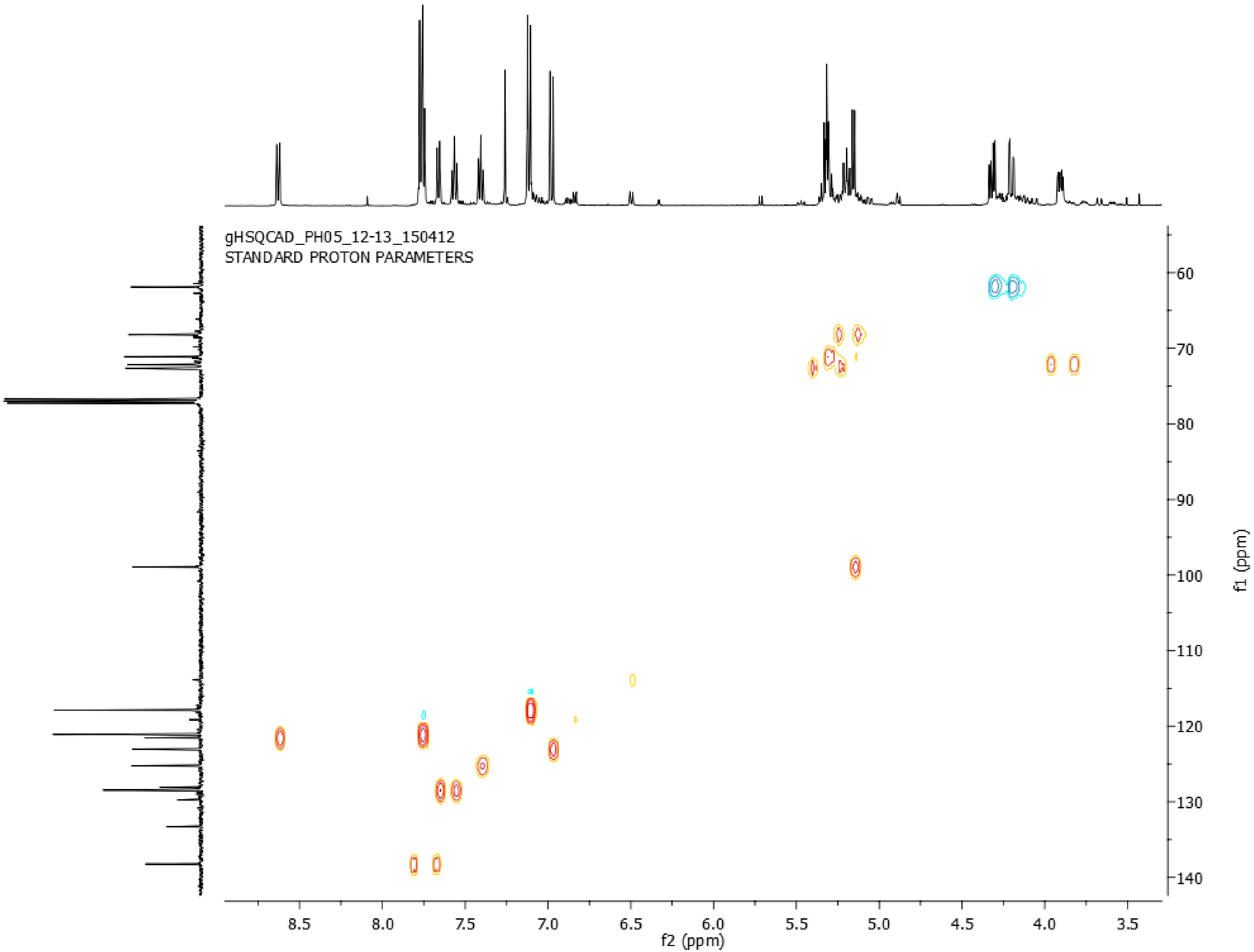
gHSQCAD_PH05_12-13_150412
STANDARD PROTON PARAMETERS
f1 (ppm)
60
70
80
90
100
110
120
130
140
8.5
8.0
7.5
7.0
6.5
6.0
5.5
5.0
4.5
4.0
3.5
f2 (ppm)

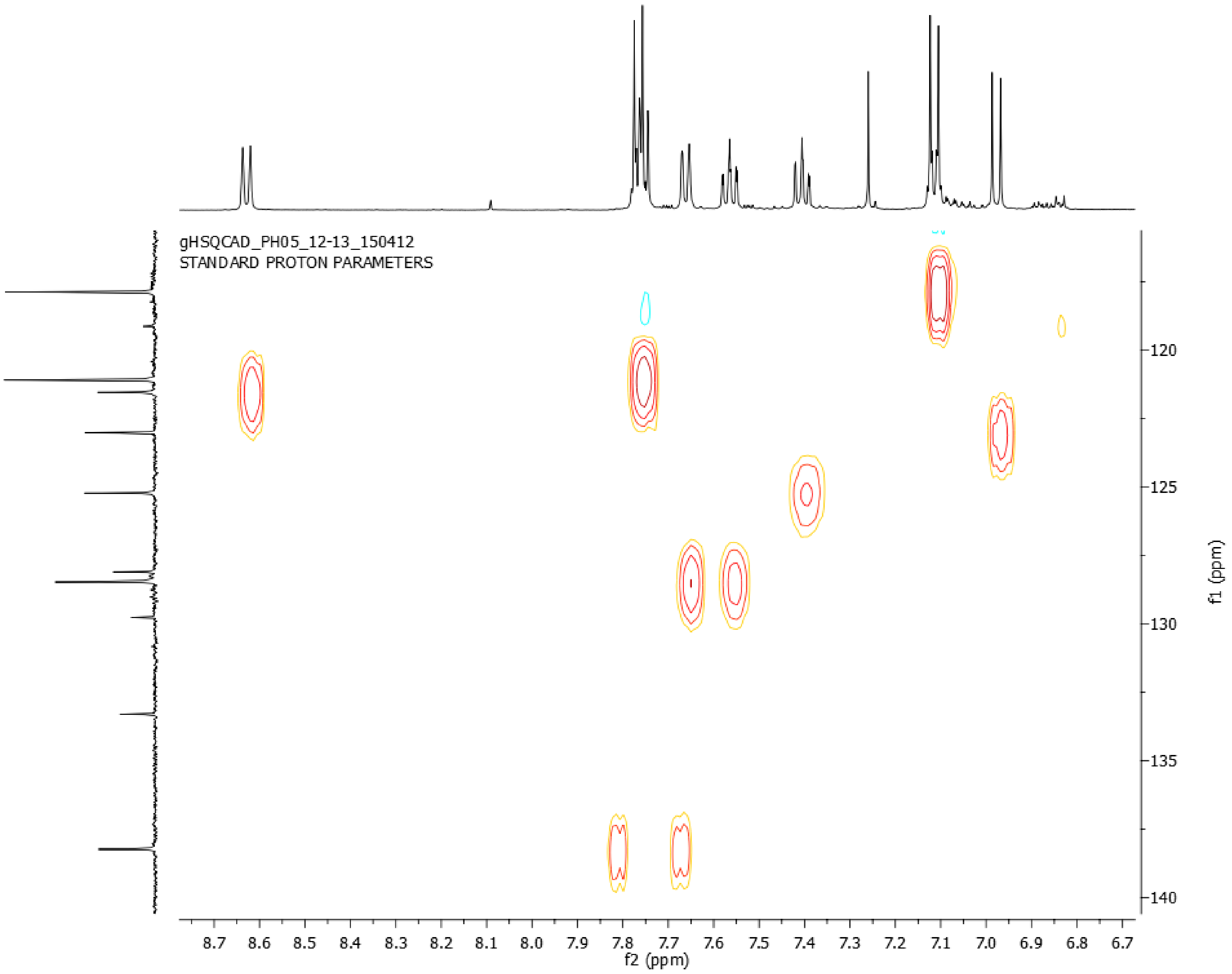

gHSQCAD_PH05_12-13_150412
STANDARD PROTON PARAMETERS
8.7 8.6 8.5 8.4 8.3 8.2 8.1 8.0 7.9 7.8 7.7 7.6 7.5 7.4 7.3 7.2 7.1 7.0 6.9 6.8 6.7
f2 (ppm)
120 125 130 135 140
f1 (ppm)

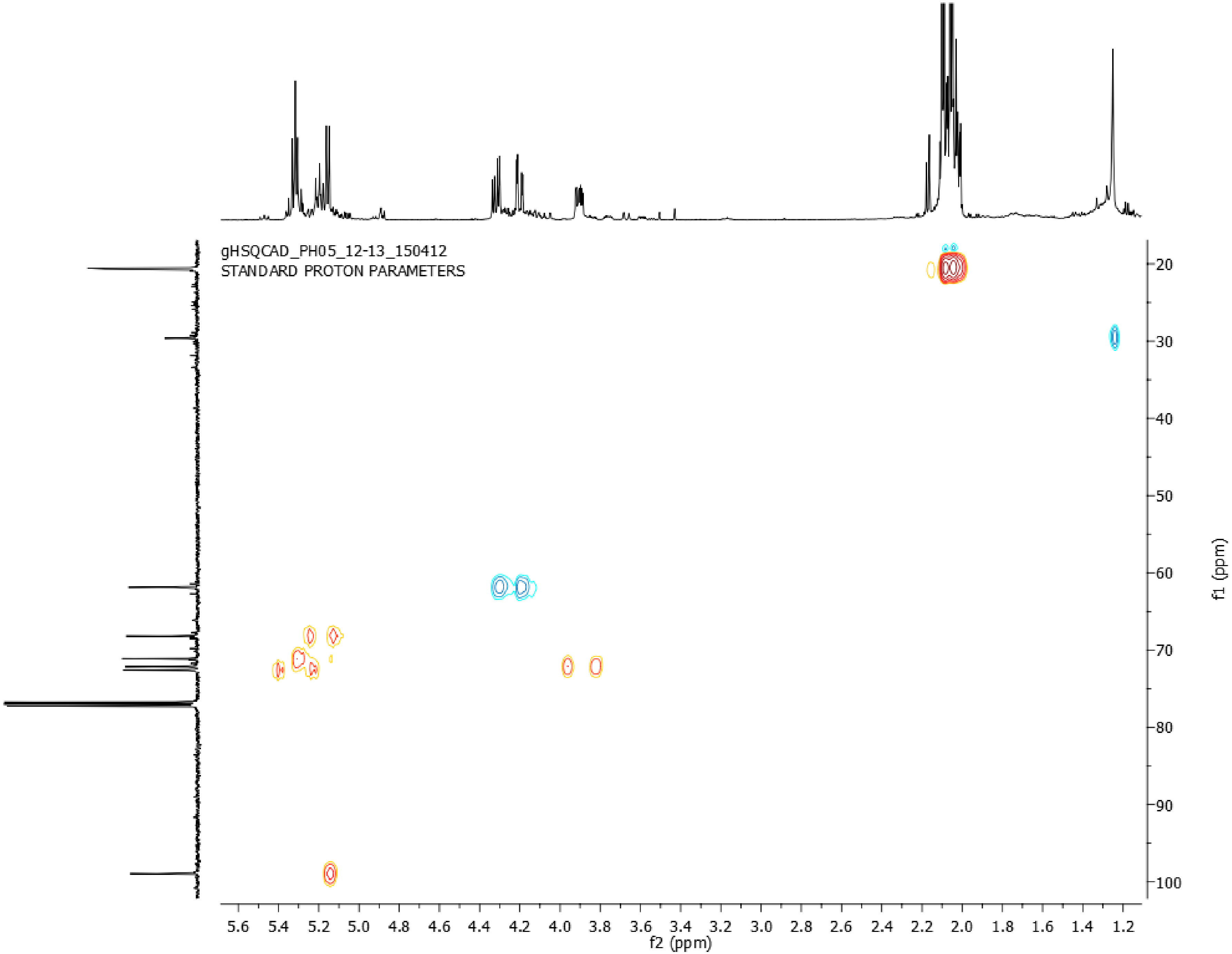
gHSQCAD_PH05_12-13_150412
STANDARD PROTON PARAMETERS
f2 (ppm)
f1 (ppm)
5.6 5.4 5.2 5.0 4.8 4.6 4.4 4.2 4.0 3.8 3.6 3.4 3.2 3.0 2.8 2.6 2.4 2.2 2.0 1.8 1.6 1.4 1.2
20 30 40 50 60 70 80 90 100

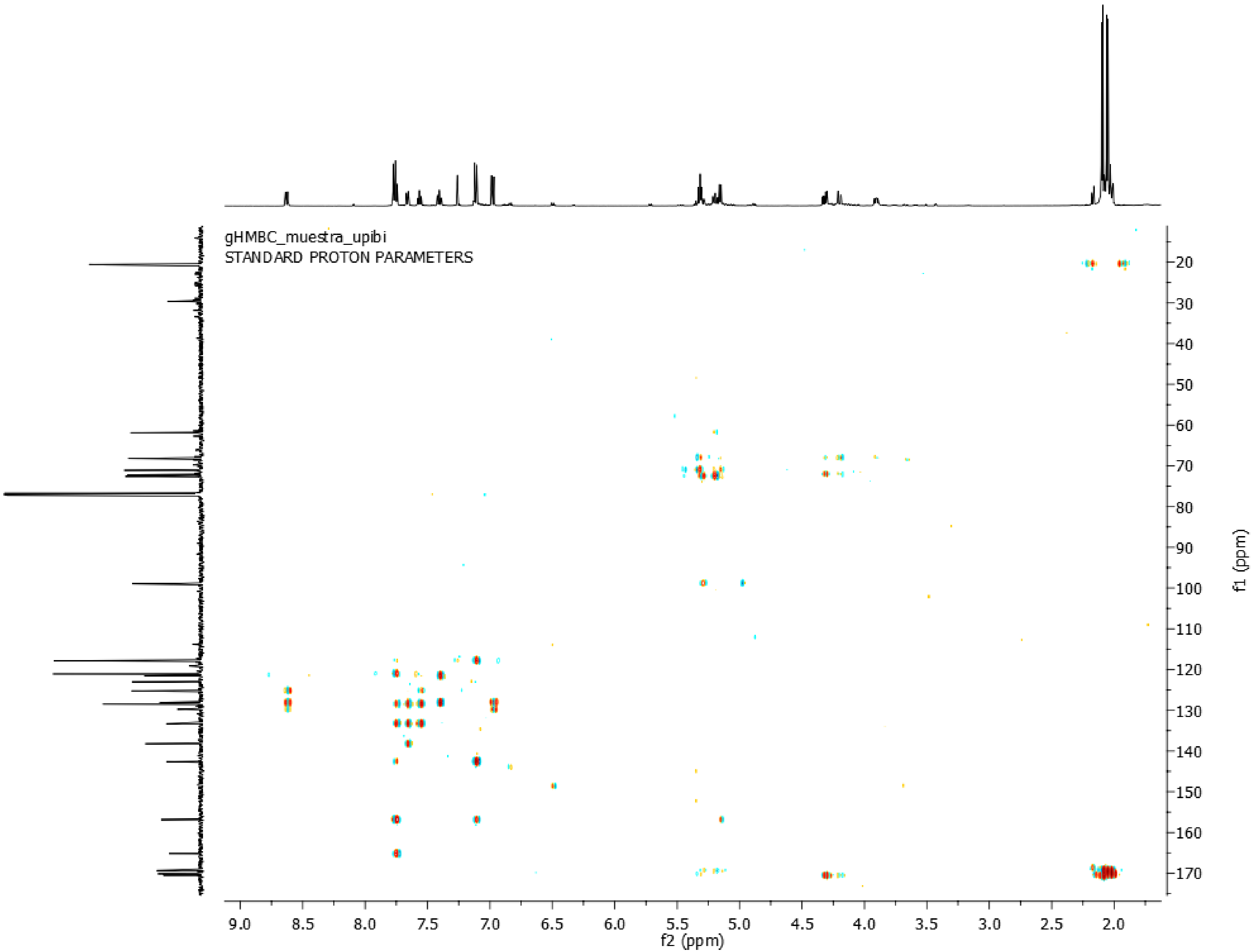
gHMBC_muestra_upibi
STANDARD PROTON PARAMETERS
f1 (ppm)
f2 (ppm)
20 30 40 50 60 70 80 90 100 110 120 130 140 150 160 170
9.0 8.5 8.0 7.5 7.0 6.5 6.0 5.5 5.0 4.5 4.0 3.5 3.0 2.5 2.0

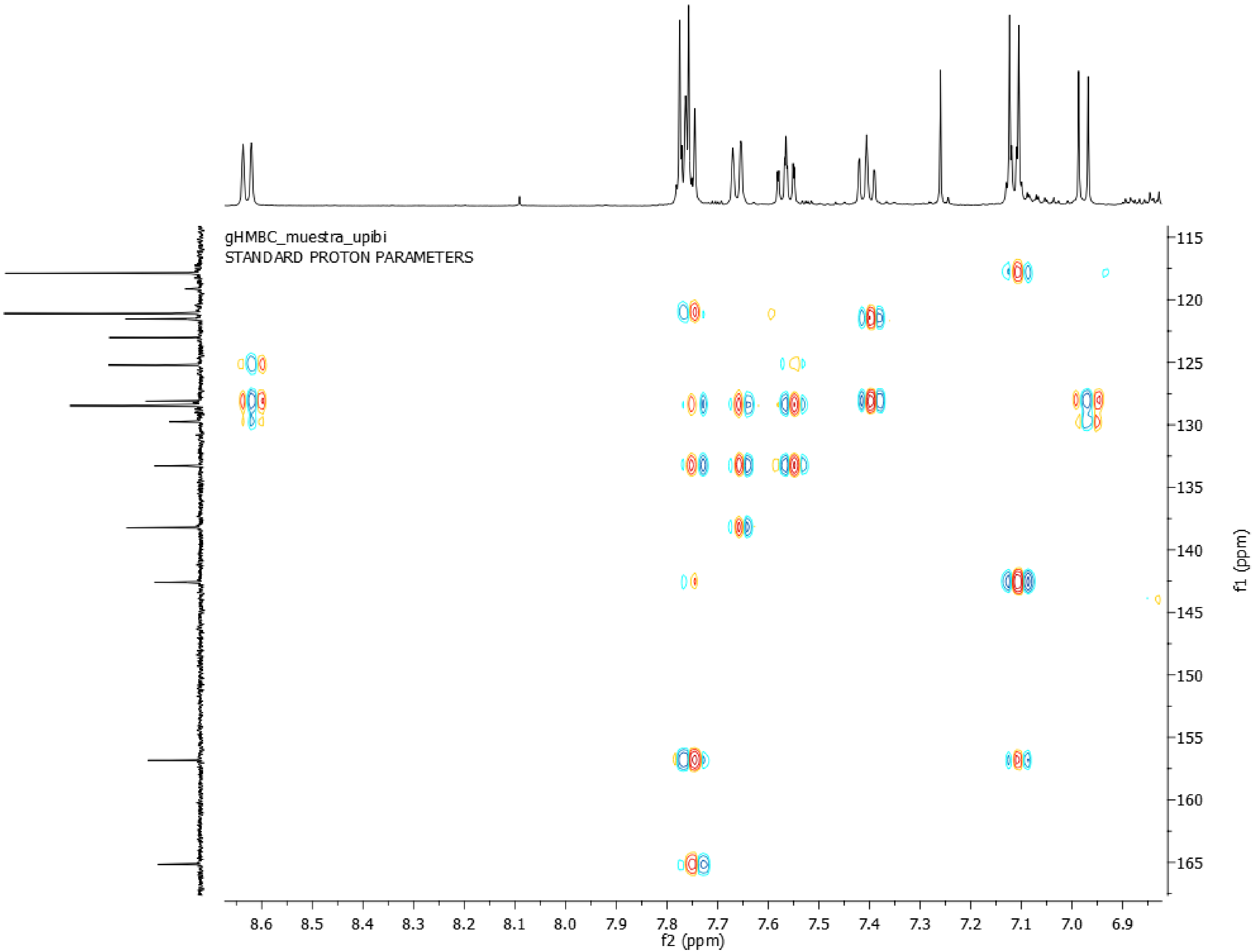
gHMBC_muestra_upibi
STANDARD PROTON PARAMETERS
f1 (ppm)
f2 (ppm)
115
120
125
130
135
140
145
150
155
160
165
8.6
8.5
8.4
8.3
8.2
8.1
8.0
7.9
7.8
7.7
7.6
7.5
7.4
7.3
7.2
7.1
7.0
6.9

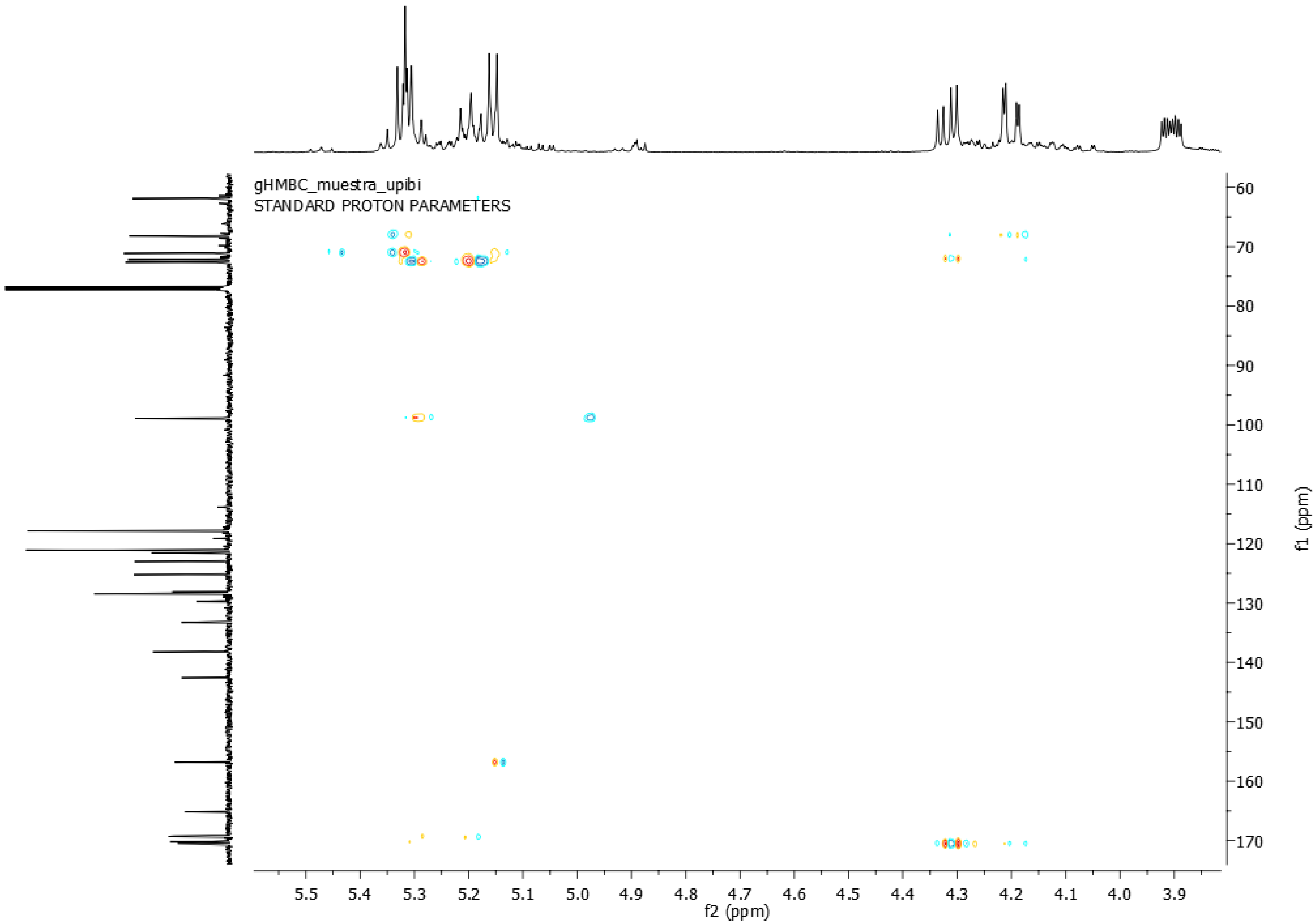
gHMBC_muestra_upibi
STANDARD PROTON PARAMETERS
f2 (ppm)
f1 (ppm)
5.5 5.4 5.3 5.2 5.1 5.0 4.9 4.8 4.7 4.6 4.5 4.4 4.3 4.2 4.1 4.0 3.9
60 70 80 90 100 110 120 130 140 150 160 170
